# Regional acidosis locally inhibits but remotely stimulates Ca^2+^ waves in ventricular myocytes

**DOI:** 10.1093/cvr/cvx033

**Published:** 2017-02-21

**Authors:** Kerrie L. Ford, Emma L. Moorhouse, Mario Bortolozzi, Mark A. Richards, Pawel Swietach, Richard D. Vaughan-Jones

**Affiliations:** 1 Burdon Sanderson Cardiac Science Centre, Department of Physiology, Anatomy and Genetics, Oxford, OX1 3PT, UK;; 2 Department of Physics and Astronomy “G. Galilei”, University of Padua, 35121 Padua, Italy

**Keywords:** Calcium cycling, Cell signalling, Membrane transport, Intracellular sodium, Intracellular pH

## Abstract

**Aims:**

Spontaneous Ca^2+^ waves in cardiomyocytes are potentially arrhythmogenic. A powerful controller of Ca^2+^ waves is the cytoplasmic H^+^ concentration ([H^+^]_i_), which fluctuates spatially and temporally in conditions such as myocardial ischaemia/reperfusion. H^+^-control of Ca^2+^ waves is poorly understood. We have therefore investigated how [H^+^]_i_ co-ordinates their initiation and frequency.

**Methods and results:**

Spontaneous Ca^2+^ waves were imaged (fluo-3) in rat isolated ventricular myocytes, subjected to modest Ca^2+^-overload. Whole-cell intracellular acidosis (induced by acetate-superfusion) stimulated wave frequency. Pharmacologically blocking sarcolemmal Na^+^/H^+^ exchange (NHE1) prevented this stimulation, unveiling inhibition by H^+^. Acidosis also increased Ca^2+^ wave velocity. Restricting acidosis to one end of a myocyte, using a microfluidic device, inhibited Ca^2+^ waves in the acidic zone (consistent with ryanodine receptor inhibition), but stimulated wave emergence elsewhere in the cell. This remote stimulation was absent when NHE1 was selectively inhibited in the acidic zone. Remote stimulation depended on a locally evoked, NHE1-driven rise of [Na^+^]_i_ that spread rapidly downstream.

**Conclusion:**

Acidosis influences Ca^2+^ waves via inhibitory $H_{i}^{\text{+}}$ and stimulatory ${\text{Na}}_{i}^{\text{+}}$ signals (the latter facilitating intracellular Ca^2+^-loading through modulation of sarcolemmal Na^+^/Ca^2+^ exchange activity). During spatial [H^+^]_i_-heterogeneity, $H_{i}^{\text{+}}$-inhibition dominates in acidic regions, while rapid ${\text{Na}}_{i}^{\text{+}}$ diffusion stimulates waves in downstream, non-acidic regions. Local acidosis thus simultaneously inhibits and stimulates arrhythmogenic Ca^2+^-signalling in the same myocyte. If the principle of remote H^+^-stimulation of Ca^2+^ waves also applies in multicellular myocardium, it raises the possibility of electrical disturbances being driven remotely by adjacent ischaemic areas, which are known to be intensely acidic.

## Introduction

An aberrant form of Ca^2+^ signalling is the Ca^2+^ wave, commonly observed in ventricular myocytes during periods of Ca^2+^-overload.^1^ Ca^2+^ waves can occur spontaneously, initiated by a localised SR Ca^2+^ release that propagates spatially, via a ‘fire-diffuse-fire’ form of Ca^2+^-induced Ca^2+^ release from the SR^2^. Ca^2+^ waves are believed to facilitate triggered arrhythmias, by driving delayed after-depolarizations (DADs) that may transition to ectopic action potentials.^3^^,^^4^ Spontaneous Ca^2+^ waves are common events during clinical conditions such as myocardial ischaemia. One factor that may trigger Ca^2+^ waves is a fall of pH_i_, although wave initiation has been variously reported to be stimulated or inhibited by acidosis.^5^^,^^6^ Ca^2+^ waves are also promoted by other factors, in particular by elevations of [Na^+^]_i_ that occur when the Na^+^/K^+^ pump is inhibited.^7^ It is notable, therefore, that a significant [Na^+^]_i_ rise occurs when the sarcolemmal Na^+^/H^+^ exchanger (NHE1) is stimulated by acidosis.^8^ In the present work, we have investigated the mechanisms coupling Ca^2+^ waves to changes of pH_i_.

Intracellular H^+^ ions are powerful modulators of cell function. In ventricular myocytes they are generated metabolically, but maintained at low cytoplasmic levels ([H^+^]_i_ ∼60 nM, equivalent to pH_i_ 7.2), most commonly via $H_{i}^{\text{+}}$ extrusion on NHE1.^9^ Despite regulation, reversible increases of [H^+^]_i_ of about 30 nM occur physiologically (equivalent to a fall of ∼0.15 pH units), while much larger increases of about 400 nM (equivalent to a fall of ∼0.6 pH units) occur during myocardial ischaemia.^10^ These [H^+^]_i_ elevations represent a form of intracellular H^+^ signalling. Prominent among the targets for such signalling are Ca^2+^ handling proteins, namely the ryanodine receptor (RyR),^11–13^ the sarco/endoplasmic reticulum Ca^2+^ ATPase (SERCA),^14^^,^^15^ Na^+^/Ca^2+^ exchanger (NCX),^16^ and the L-type calcium channel.^17^ Although direct H^+^ interaction with these proteins is predominantly inhibitory, an elevation of [H^+^]_i_ can *enhance* the electrically evoked calcium transient (CaT),^18^ an effect that helps to protect contractility during acidosis. As mentioned above, this latter stimulation occurs indirectly through H^+^ activation of NHE1. The resulting rise of [Na^+^]_i_ slows Ca^2+^ efflux on sarcolemmal NCX, which boosts sarcoplasmic reticulum (SR) Ca^2+^ loading via SERCA, thereby increasing CaT amplitude.^18^^,^^19^ Quite how the balance between inhibitory and excitatory effects of $H_{i}^{\text{+}}$ on Ca^2+^ signalling impacts on the generation of arrhythmogenic Ca^2+^ waves has yet to be examined.

The importance of *spatial* interactions among [H^+^]_i_, [Na^+^]_i_ and [Ca^2+^]_i_ in ventricular myocytes has been highlighted in recent work.^18^ Because of high intracellular buffering, cytoplasmic H^+^ mobility is low,^20^ so that localised [H^+^]_i_ microdomains can form.^21^ A microdomain of elevated [H^+^]_i_, by locally stimulating NHE1, elevates ${\text{Na}}_{i}^{\text{+}}$, which increases the CaT amplitude. But because Na^+^ diffuses rapidly in cytoplasm,^18^^,^^22^ it can enhance the CaT globally within the cell, including in regions not experiencing acidosis. In the present work we investigate if spontaneous Ca^2+^ waves are similarly controlled through interacting spatial $H_{i}^{\text{+}}$ and ${\text{Na}}_{i}^{\text{+}}$ signals.

In order to investigate the coupling of Ca^2+^ waves to pH_i_, we have manipulated intracellular pH, Na^+^, and Ca^2+^ in rat ventricular myocytes. Our data indicate that H^+^ ions exert direct inhibitory and indirect excitatory effects on Ca^2+^ waves. The excitatory effect is mediated via intracellular Na^+^. When excitation is induced by a localised acidic microdomain, it is typically expressed remotely in non-acidic regions of the cell, driven by fast ${\text{Na}}_{i}^{\text{+}}$ diffusion. Our data provide evidence that acidosis, and its spatial heterogeneity, is a powerful substrate for the initiation and spatial organisation of pro-arrhythmic Ca^2+^ waves.

## Methods

Detailed methods are available in the Supplementary material online.

### Ventricular myocyte isolation

All procedures were performed in accordance with UK Home Office and local guidelines. Ventricular myocytes were isolated from 46 male Sprague-Dawley rats, as previously described,^23^ or from neonatal rats (1 day old).

### Fluorescence measurements of intracellular pH and Ca^2+^

*pH_i_*: myocytes were loaded with carboxy-seminaphthorhodofluor-1 (cSNARF-1, 10 μM) at room temperature. Cells were imaged confocally on a Leica SP5 inverted microscope. Intracellular calibration of cSNARF-1 was performed in separate experiments using nigericin.^23^

*Ca^2+^ waves and sparks*: myocytes were AM-loaded with fluo-3 (18 μM). Cells were superfused and quiescent cells were imaged in linescan (*xt*) mode along the cell’s longitudinal axis at 400 lines per second. Ca^2+^ sparks were imaged in solution containing 1 mM free Ca^2+^. Switching the superfusate to one containing raised (5 mM) free Ca^2+^ induced a modest calcium overload that triggers Ca^2+^ waves.

### Generating intracellular pH gradient

A pH_i_ gradient was induced using a microfluidic device consisting of a square-bore double-barrelled micropipette, which released two parallel microstreams of solution perpendicular to the cell.^24^ One microstream contained Tyrode with 5 mM CaCl_2_ plus 10 mM sucrose to help visualize the interstream boundary; the other microstream contained 80 mM acetate with 5 mM free Ca^2+^. The position of the microstream boundary across the cell was recorded in *xy* before and after each linescan experiment. The smooth pH_i_ gradient induced is illustrated in *Figure 4B*.

### Data analysis

Data are expressed as mean ± SE. Statistical significance was tested using paired Student’s *t*-test or nested ANOVA, depending on the experimental protocol. Where relevant, the Holm correction for multiple testing was applied. Significance is denoted as * for *P *<* *0.05, ** for *P *<* *0.01, *** for *P *<* *0.001.

Wave propagation velocity was calculated from linescan images using the angle of incidence of the wavefront, relative to the longitudinal axis of the cell.

Linescan recordings of Ca^2+^ sparks were normalized using a custom-built Matlab macro, followed by spark frequency analysis using an algorithm developed by Kong *et al.*^25^

## Results

### Acidosis affects Ca^2+^ waves via $H_{i}^{\text{+}}$ and ${\text{Na}}_{i}^{\text{+}}$ signals

**Figure 1:**
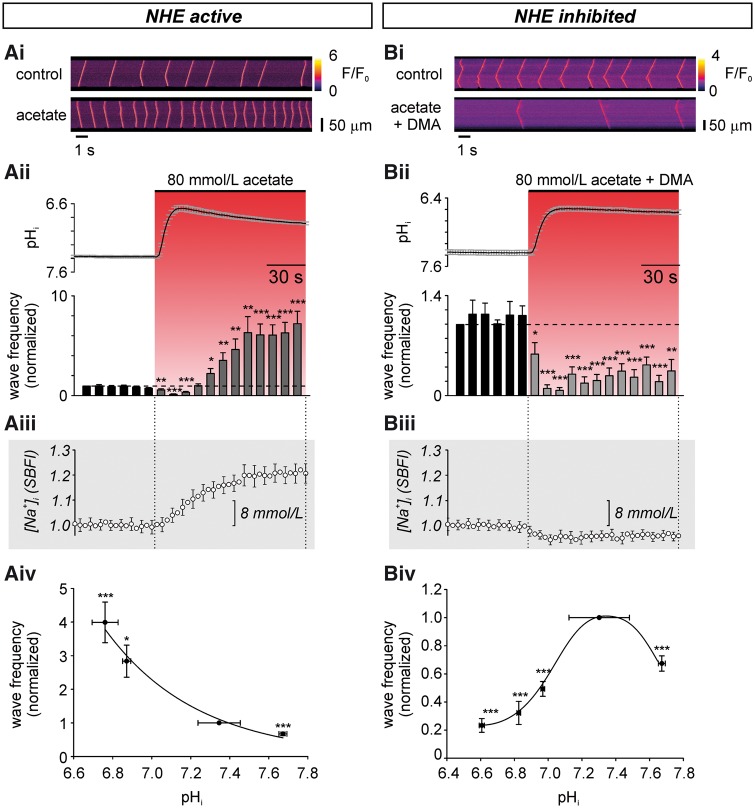
Intracellular acidosis stimulates Ca^2+^ waves in an NHE1-dependent manner. (*Ai*) Ca^2+^ waves (fluo-3) were triggered at 5 mM extracellular [Ca^2+^]. Images recorded in linescan (xt) mode along the longitudinal axis of the cell at 400 lines per second. F/F_0_ = fluorescence divided by resting fluorescence (F_0_ averaged over first 1 s of recording). (*Aii*) Decreasing pH_i_ (cSNARF-1, mean pH_i_ trace, error bars are SEM, *n* = 13 cells/2 animals) by 80 mM acetate superfusion initially decreased, then increased wave frequency (*n* = 18 cells/5 animals). Waves were counted in 10 s bins and normalized to the mean frequency in the first 10 s bin. (*Aiii*) [Na^+^]_i_ timecourse, on the same timescale as Aii and taken under comparable experimental conditions, replotted from *Figure 2B* of reference 18. SBFI ratio acquired every 4 s, *n* = 6 cells/2 animals, error bars are SEM. (*Aiv*) Ca^2+^ wave frequency showed a positive relationship with decreasing pH_i_. Error bars are SD (pH_i_), SEM (frequency). Wave frequency and pH_i_ averaged over 1 min, from 30 s after onset of acetate superfusion. For 80 mM acetate *n* = 18 cells/5 animals (waves), 13 cells/2 animals (pH_i_); 40 mM acetate *n* = 5 cells/1 animal (waves), 8 cells/2 animals (pH_i_); 20 mM TMA (for extreme intracellular alkalosis) *n* = 10 cells/2 animals (waves), 8 cells/2 animals (pH_i_). (*Bi*) Ca^2+^ wave frequency during acidosis with NHE1 inhibitor (30 μM DMA). (*Bii*) NHE1 inhibition attenuates pH_i_ recovery during acidosis (*n* = 13 cells/3 animals) and reveals an underlying inhibitory effect of intracellular acidosis on Ca^2+^ wave frequency (*n* = 12 cells/3 animals). (*Biii*) [Na^+^]_i_ timecourse, on the same timescale as Bii and taken under comparable experimental conditions, replotted from *Figure 2B* of reference 18. SBFI ratio acquired every 4 s, *n* = 7 cells/2 animals, error bars are SEM. (*Biv*) Relationship between wave frequency and pH_i_ in absence of NHE1 activity. Error bars are SD (pH_i_), SEM (frequency). For 80 mM acetate + DMA *n* = 12 cells/3 animals (waves), 17 cells/3 animals (pH_i_); 40 mM acetate + DMA *n* = 9 cells/2 animals (waves), 6 cells/2 animals (pH_i_); 20 mM acetate + DMA *n* = 9 cells/2 animals (waves), 8 cells/2 animals (pH_i_); 20 mM TMA *n* = 10 cells/2 animals (waves), 8 cells/2 animals (pH_i_). Paired *t*-tests.

**Figure 2:**
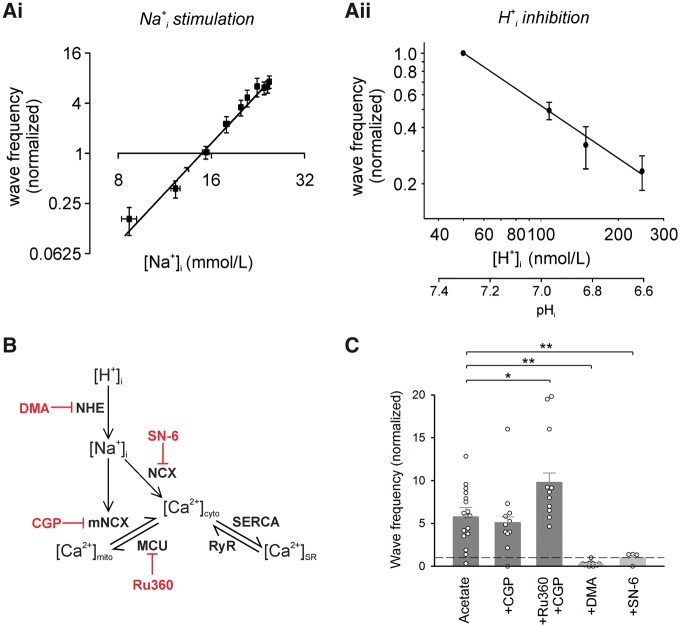
Acidosis-induced stimulation of Ca^2+^ waves depends on sarcolemmal NCX. (*Ai*) Ca^2+^ wave initiation frequency is steeply dependent on [Na^+^]_i_. Wave frequency ∝ [Na^+^]_i4_. Note the logarithmic axes. (*Aii*) In the absence of an NHE-driven [Na^+^]_i_ rise, Ca^2+^ waves show a linear, inverse relationship with [H^+^]_i_. Note the logarithmic axes. Fit using linear regression: 50.5 × [H^+^]^−1^. (*B*) Proposed interactions between intracellular H^+^, Na^+^, and Ca^2+^ modify SR Ca^2+^ load, and thus Ca^2+^ wave probability. Pharmacological inhibitors are in red. (*C*) Wave frequency averaged during 2nd min of acetate superfusion. Inhibiting mitochondrial NCX (‘CGP’: 20 μM CGP-37157) had no effect on acidosis-induced Ca^2+^ wave stimulation (*n* = 11 cells/2 animals). Inhibiting both mitochondrial NCX and the mitochondrial Ca^2+^-uniporter (‘Ru360’: 10 μM ruthenium-360) enhanced wave stimulation (*n* = 11 cells/5 animals). Inhibiting sarcolemmal NCX (10 μM SN-6) prevented acidosis-induced wave stimulation (*n* = 4 cells/2 animals). Nested ANOVA followed by pairwise comparison with Holm correction for multiple testing.

We explored the $H_{i}^{\text{+}}$-sensitivity of Ca^2+^ waves, by first imposing whole-cell changes of pH_i,_ at constant pH_o_. Individual ventricular myocytes, when subjected to modest Ca^2+^ overload (5 mM ${\text{Ca}}_{o}^{2\text{+}}$), displayed spontaneous Ca^2+^ waves (*Figure 1Ai*), with a frequency of 18.7 ± 2.5 min^−1^. At times these waves converted into whole-cell CaTs (Supplementary material online, *Figure S1*), induced by the Ca^2+^-driven transient inward current (*I*_ti_) triggering an action potential.^3^^,^^4^ Cells displaying wave-triggered CaTs were not included in the analysis, because of their confounding effects on wave properties. Intracellular acidification (from pH_i_ 7.3 to 6.6), induced by superfusion of 80 mM acetate, initially reduced wave frequency (*Figure 1Aii*). After 30 s, wave frequency then progressively increased above control levels (*Figure 1Ai, ii*, Supplementary material online, *FigureS2A*). The increase could be graded by varying the magnitude of intracellular acidosis (superfusion with 20, 40, or 80 mM acetate; *Figure 1Aiv*). The increase was attributable to an $H_{i}^{\text{+}}$-dependent stimulation of NHE1 activity, as it was abolished in the presence of 5-(*N*,*N*-dimethyl) amiloride (DMA, 30 μM), a high-affinity NHE1 inhibitor. Under these conditions, reducing pH_i_ from 7.3 to 6.6 now produced a decrease in Ca^2+^ waves (*Figure 1Bi, ii*, Supplementary material online, *FigureS2B*), which was also graded with the severity of acidosis (*Figure 1Biv*; note that, in these latter experiments, extreme alkalosis, produced by 20 mM trimethylamine superfusion, also reduced wave frequency). Abolition of $H_{i}^{\text{+}}$-evoked Ca^2+^ waves was confirmed with a structurally different NHE1 inhibitor, cariporide, which targets NHE1 more specifically among NHE isoforms than DMA^26^; Supplementary material online, *Figure S2C, D*. Thus, for the pH_i_ range from 7.3 to 6.6, raising [H^+^]_i_ exerts opposing effects on Ca^2+^ wave frequency; an NHE1-independent *inhibition*, and a delayed, NHE1-dependent *stimulation*.

H^+^-evoked stimulation of Ca^2+^ waves is accompanied by a DMA-sensitive rise of [Na^+^]_i_ (*cf*. *Figures 1Aiii*, *1Biii*), consistent with enhanced Na^+^ influx on NHE1. The [Na^+^]_i_ rise leads, via a slowing of Ca^2+^ efflux on sarcolemmal NCX, to retention of intracellular Ca^2+ 7^ and, ultimately, to increased Ca^2+^ wave frequency. The [Na^+^]_i_ measurements have been extracted from our previously published work, measured under comparable experimental conditions^18^ (as the published [Na^+^]_i_-rise was recorded in 1 mM [Ca^2+^]_o_, whereas current experiments were conducted in 5 mM [Ca^2+^]_o_, we confirmed that the rise was not influenced by the different [Ca^2+^]_o_ levels; see Supplementary material online, *Figure S6*). For clarity, [Na^+^]_i_ data have been replotted in *Figures 1Aiii and 1Biii*. The data emphasize that preventing the H^+^-evoked rise of [Na^+^]_i_ with DMA, prevents wave stimulation. Stimulation is thus dependent on the rise of [Na^+^]_i_, while acid-induced wave inhibition, observed in the presence of an NHE1 inhibitor, is attributable directly to the rise of [H^+^]_i_. *N.B*. since the rise of [Na^+^]_i_ is completely prevented by inhibiting NHE, we do not anticipate a role for H^+^-induced inhibition of the Na^+^/K^+^ pump under these conditions.

Ca^2+^ wave stimulation has been quantified in *Figure 2Ai*, which plots, on logarithmic axes, wave frequency vs. [Na^+^]_i_ (data amalgamated from *Figure 1Aii, Aiii*). Wave frequency rises steeply with [Na^+^]_i_ (to a first approximation, wave frequency ∝ [Na^+^]_i4_). In contrast, wave frequency in the absence of NHE1 activity *decreased* with a rise of [H^+^]_i_, displaying a more shallow H^+^-dependence (*Figure 2Aii*). The net result for whole-cell acidosis on wave frequency will be a combination of these stimulatory and inhibitory components. At steady-state, the combination is net stimulatory (*Figure 1Aii, iv*), reflecting the steep positive dependence of wave frequency on [Na^+^]_i_.

### Mitochondrial NCX does not promote Ca^2+^ waves during acidosis

Although sarcolemmal NCX is likely to mediate the [Na^+^]_i_-dependence of Ca^2+^ wave frequency, this does not exclude an additional role for mitochondrial NCX (mNCX). Elevating [Na^+^]_i_ has been proposed to promote mitochondrial Ca^2+^ efflux on mNCX,^27^ in addition to slowing the forward mode of sarcolemmal NCX. Promoting mitochondrial Ca^2+^ efflux may therefore supplement the domain of cytoplasmic Ca^2+^ that eventually enhances Ca^2+^ wave frequency. To test this hypothesis, myocytes were first subjected to intracellular acidosis in the presence of the sarcolemmal NCX inhibitor, SN-6. The drug fully inhibited the H^+^-evoked stimulation of Ca^2+^ wave frequency (*Figure 2C*), indicating a necessary role for the sarcolemmal exchanger. In contrast, selective inhibition of mNCX with CGP-37157 had no effect on H^+^-evoked waves (*Figure 2C*), indicating that mNCX is unlikely to be supplementing Ca^2+^ wave stimulation. Indeed, it is more likely that mitochondria attenuate Ca^2+^ waves. This is supported by the observation that when the mitochondrial uniporter (MCU), which mediates Ca^2+^ uptake, was blocked by pre-incubation with ruthenium-360 (Ru360), followed by inhibition of mNCX with CGP-37157, then H^+^-induced Ca^2+^ wave frequency was *enhanced* by 70% (*Figure 2C*). The result suggests that mitochondrial Ca^2+^ uptake normally plays a protective role during intracellular acidosis, by sequestering some of the intracellular Ca^2+^-overload.

### Acidosis affects multiple properties of Ca^2+^ waves

**Figure 3:**
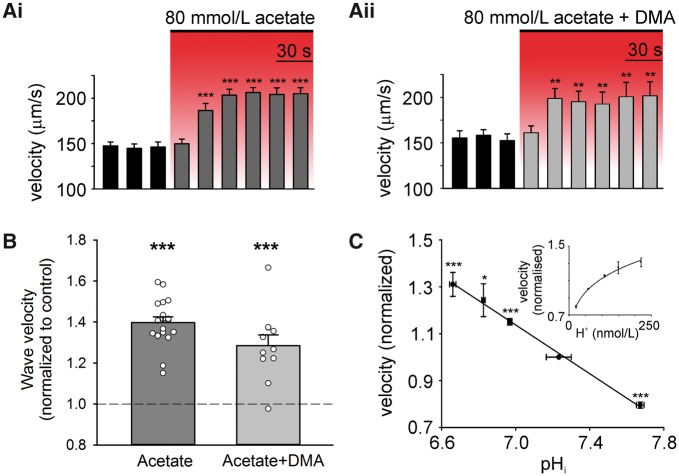
Intracellular acidosis alters Ca^2+^ wave velocity. (*A*) Intracellular acidosis increases velocity of Ca^2+^ waves in the presence (*n* = 18 cells/5 animals) (*i*) and absence (i.e. with 30 µM DMA; *n* = 11 cells/2 animals) (*ii*) of NHE1 activity. (*B*) Normalized wave velocity measured during 2nd min of 80 mM acetate superfusion*.* (*C*) Wave velocity and pH_i_ were averaged over 100 s, starting 20 s after onset of acetate superfusion with 30 μM DMA (80 mM acetate + DMA *n* = 11 cells/2 animals (waves), 5 cells/1 animals (pH_i_); 40 mM acetate + DMA *n* = 4 cells/1 animal (waves), 6 cells/2 animals (pH_i_); 20 mM acetate + DMA *n* = 8 cells/2 animals (waves), 8 cells/2 animals (pH_i_); 20 mM TMA *n* = 12 cells/2 animals (waves), 8 cells/2 animals (pH_i_)). Fit: *y*=-0.5223*x *+* *4.7943 (*R*^2^ = 0.996). Paired *t*-tests.

In addition to Ca^2+^ wave frequency, we investigated whether raising [H^+^]_i_ influenced other features, notably, Ca^2+^ wave propagation velocity (v_prop_), amplitude, and time-course. Raising [H^+^]_i_ increased wave velocity, both in the presence and absence of NHE1 activity (*Figure 3A, B*). As shown in *Figure 3C*, velocity rose with a fall of pH_i_ from 7.7 to 6.65 (equivalent to a 250 nM rise in [H^+^]_i_), increasing by ∼40% over the range. According to a recent model of Ca^2+^ wave propagation,^28^ a plausible explanation for the velocity increase is an H^+^-dependent reduction in cytoplasmic Ca^2+^ buffering, for example, by troponin C and other proteins, as well as by cytoplasmic histidyl-dipeptides.^24^ Decreased Ca^2+^ buffering may reflect a reduction in the Ca^2+^ binding constant (k_on_), or an increase in the unbinding constant (k_off_). Increasing [H^+^]_i_ also increased resting [Ca^2+^]_i_ (as reported previously^24^) and peak F/F_0_ fluorescence, and slowed Ca^2+^ wave relaxation (Suuplementary material online, *Figure S3*).

### Local acidosis stimulates downstream Ca^2+^ waves in remote regions of the myocyte

**Figure 4:**
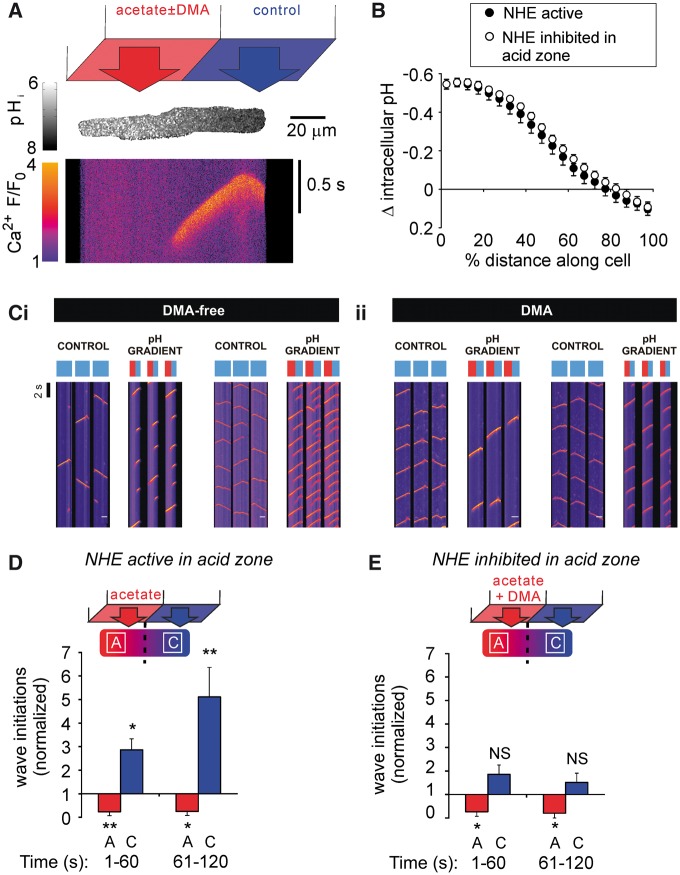
An acidic microdomain affects Ca^2+^ wave initiation both locally and remotely. (*A*) A longitudinal pH_i_ gradient was generated by regional superfusion of the myocyte with 80 mM acetate. Top: schematic of dual microperfusion apparatus; middle; representative pH_i_ gradient along a myocyte, *xy* image with SNARF-1; bottom; representative Ca^2+^ wave recorded in linescan mode during an imposed pH_i_ gradient. (*B*) Magnitude of pH_i_ gradient (*n* = 12 cells/4 animals) was unaffected by inhibiting NHE1 in the acidic microdomain (*n* = 11 cells/2 animals). (*Ci*) Exemplar linescans recorded from 2 rat ventricular myocytes, under control conditions (left panel of each cell), and during imposition of a longitudinal pH_i_ gradient by regional superfusion of the myocyte with 80 mM acetate (right panel of each cell). In order to compress the results, the timecourse for the line-scans has been broken into three parallel columns. Note that, during control conditions, Ca^2+^ waves initiate randomly along the length of the myocyte, whereas, during imposition of the pH_i_ gradient, waves initiate almost exclusively in the non-acidic microdomain (represented by the blue bar above each column of line-scans), and their frequency increases. The acidic zone is represented by the red bar above each column of linescans. (*Cii*) Exemplar linescans recorded from 2 cells under equivalent conditions, but with 30 µM DMA included in the regional acetate superfusion (to inhibit NHE activity). During imposition of the pH_i_ gradient, wave initiations remain restricted to the non-acidic microdomain, but now their overall frequency does not change compared with control. (*D*) Ca^2+^ wave initiation was inhibited in the acidic (acetate, A) microdomain and remotely stimulated in the non-acidic (control, C) microdomain (*n* = 6 cells/3 animals). (*E*) Ca^2+^ wave initiation was attenuated in the acidic microdomain, but absence of NHE1 activity prevented the remote stimulation of Ca^2+^ waves in the non-acidic microdomain (*n* = 5 cells/2 animals). Paired *t*-tests.

Acidosis was imposed locally at one end of an isolated myocyte using a dual micro-superfusion device positioned at right-angles to the cell (see Methods). One microstream contained 80 mM acetate while the other contained acetate-free Tyrode (*Figure 4A*). This generated a stable end-to-end pH_i_ gradient of ∼0.6 units (*Figure 4B*).

Under control conditions (before imposing the pH_i_ gradient), no significant difference was observed in Ca^2+^ wave frequency between ROIs positioned at opposite ends of the cell (*P *=* *0.23; these regions were defined as segments along the line-scan at either end of the myocyte, of length equal to one third of total cell length). In contrast, after imposing the pH_i_ gradient, Ca^2+^ waves were initiated, and became clustered in the non-acidic end of the cell, even though this region was many sarcomere-lengths away from the acidic zone. Thus, over the first 2 min of an imposed pH_i_ gradient, Ca^2+^ waves were rarely initiated in the acidic zone, but their frequency increased up to five-fold in the non-acidic zone (*Figure 4Ci, D*). Selective pharmacological inhibition (DMA) of NHE1 activity in the non-acidic zone did not prevent the local increase in Ca^2+^ wave frequency (*P *=* *0.01, *n* = 6), indicating that the relevant NHE1 activity driving wave initiation was in the acidic zone. This was tested further by selectively superfusing DMA over the acidic end of the myocyte. Although the pH_i_ gradient itself was unaffected by this manoeuvre, the stimulation of Ca^2+^ waves in the non-acidic zone was now greatly attenuated (*Figure 4Dcf. 4E*). Thus, locally enhancing NHE1 activity remotely stimulates Ca^2+^ waves.

In summary, during uniform acidosis, all regions of a myocyte are equally likely to support a Ca^2+^ wave, driven by NHE1 activity. However, when NHE1 is stimulated *locally* (by inducing a localised acidosis within the cell) there is selective stimulation and clustering of Ca^2+^ waves in *downstream non-acidic zones*.

### Local acidosis inhibits local Ca^2+^ sparks

Since H^+^ ions affect multiple proteins involved in Ca^2+^ signalling, the mechanism by which Ca^2+^ waves are inhibited in the acidic microdomain, but remotely stimulated in the non-acidic microdomain, is not immediately obvious. One possibility is that H^+^ ions locally decrease RyR open probability (P_o_), which could account for inhibition of wave initiation. A decreased P_o_ may also enhance SR Ca^2+^ retention.^29^ This could contribute to remote wave stimulation if the locally elevated SR Ca^2+^ load then diffused rapidly into non-acidic regions of the SR lumen.

**Figure 5:**
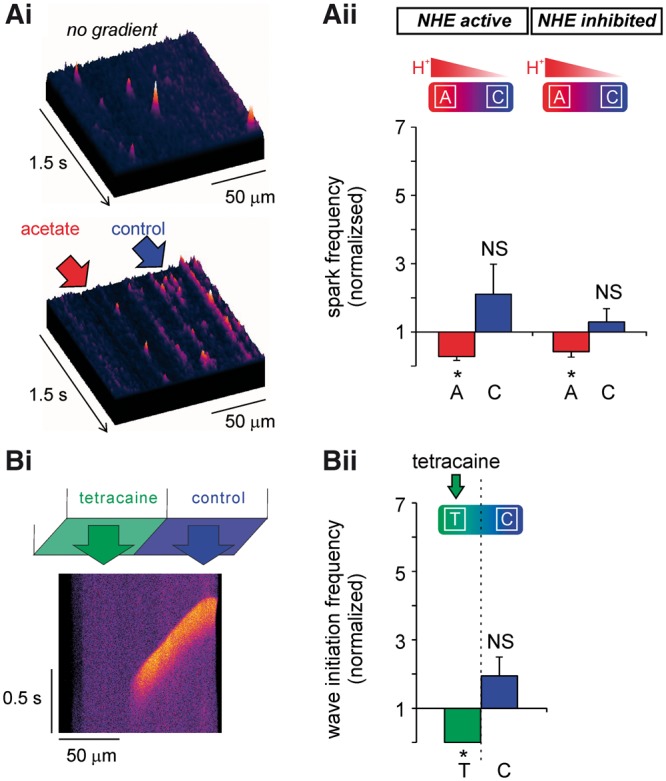
Inhibition of RyR channels by H^+^ ions can account for regional Ca^2+^ wave inhibition during pH_i_ heterogeneity. (*Ai*) 3D surface plots of Ca^2+^ sparks (recorded in linescan mode) illustrate a uniform distribution under resting conditions. Imposed pH_i_ gradient inhibits spark events in acidic (acetate, A) microdomain. (*Aii*) Sparks were inhibited in the acidic microdomain in the presence (*n* = 9 cells/2 animals) and absence (*n* = 5 cells/1 animal) of NHE1 activity. (*Bi*) Top panel represents dual microperfusion apparatus. Linescan of a Ca^2+^ wave during regional superfusion with tetracaine. (*Bii*) Wave initiation and propagation were suppressed in the tetracaine-exposed microdomain (remote effect is not significant; *n* = 12 cells/3 animals, *P *=* *0.1175). Paired *t*-tests.

To explore whether the spatial effects of acidosis on wave frequency are all due to RyR inhibition, Ca^2+^ spark frequency was measured. Under resting conditions, spark frequency at either end of the myocyte was not different (*P *=* *0.33, *n* = 9). However, when a pH_i_ gradient was imposed, Ca^2+^ spark frequency in the acidic microdomain decreased by 72 ± 11% (*P *=* *0.0002, *n* = 9), while that in the non-acidic microdomain was not significantly altered (*Figure 5A*, *P *=* *0.24, *n* = 9). This phenomenon was insensitive to NHE1 inhibition (*Figure 5Aii*). The results argue for local H^+^ inhibition of RyRs, but also indicate that any resulting local rise in luminal SR [Ca^2+^] does not affect the P_o_ of remote RyRs.

To verify the effect on Ca^2+^ waves of regional RyR inhibition, tetracaine (an RyR antagonist) was applied to one half of a myocyte to simulate the inhibitory effect of H^+^ ions, while maintaining pH_i_ uniformly at resting levels throughout the cell. *Figure 5B* shows that Ca^2+^ wave initiation frequency observed with locally applied tetracaine was very similar to that observed with local acidosis and NHE1 inhibition (*cf.Figure 4E*). In both cases, Ca^2+^ wave frequency was suppressed in the tetracaine/acetate-exposed region of the cell, and did not significantly change in the downstream (unexposed) region. These findings confirm that, while local RyR blockade by H^+^ ions or tetracaine can explain local (upstream) inhibition of Ca^2+^ waves, it cannot explain wave stimulation in downstream regions. This latter effect requires the local, upstream activity of NHE1.

### Properties of the Ca^2+^ wave map onto pH_i_ gradients

**Figure 6:**
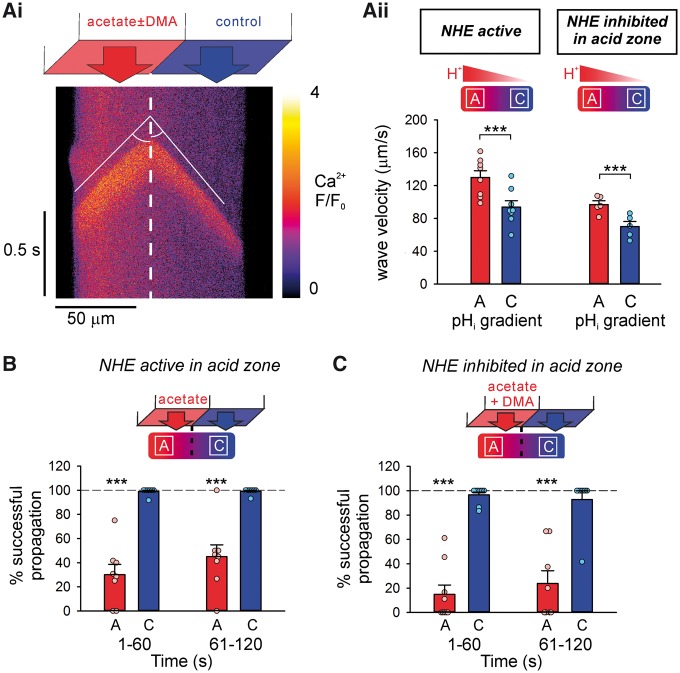
Ca^2+^ wave velocity and propagation map on to local pH_i_ non-uniformities. (*Ai*) During a stable longitudinal pH_i_ gradient, Ca^2+^ wave is faster in the acidic (acetate) microdomain. (*Aii*) Wave propagation velocity in the acidic (acetate, A) and non-acidic (control, C) microdomains in the presence (*n* = 8 cells/4 animals) or absence (*n* = 5 cells/3 animals) of NHE1 activity. (*B*) Wave propagation was suppressed in the acidic microdomain, while remaining unaffected in the non-acidic microdomain (*n* = 8 cells/4 animals). (*C*) NHE1 inhibition had no effect on the success of wave propagation in the acidic microdomain (*n* = 9 cells/2 animals). Dashed lines represent control. Paired *t*-tests.

To investigate whether properties of Ca^2+^ waves, other than initiation frequency, also map onto pH_i_ heterogeneity, Ca^2+^ wave velocity (v_prop_), amplitude and time-course were measured in the acidic and non-acidic microdomains. For the few Ca^2+^ waves that successfully propagated from the non-acidic to the acidic zones of the cell, their velocity increased in the acidic zone (*Figure 6Aii*, 40 ± 7% faster, *P *=* *0.0002, *n* = 8). A similar trend was observed when DMA was superfused over the acidic end of the cell, indicating that the faster propagation was independent of NHE1 activity (*Figure 6Aii*, 40 ± 6% faster v_prop_ than non-acidic microdomain, *P *=* *0.0002, *n* = 5).

The peak F/F_0_ of the Ca^2+^ wave was also significantly higher in the acidic microdomain compared with the non-acidic microdomain (Supplementary material online, *Figure S4*), while wave relaxation was significantly slower in the acidic microdomain (Supplementary material online, *Figure S4*). It was also noted that Ca^2+^ waves frequently failed to propagate within the acidic microdomain (irrespective of the initiation site), unlike the negligible failure rate observed under control conditions (*Figure 6B, C*). Taken together, our results show that multiple properties of the Ca^2+^ wave (velocity, propagation-failure, amplitude, and relaxation rate, in addition to initiation frequency) are all subservient to the local pH_i_. Spatial non-uniformity of pH_i_ thus induces dramatic heterogeneity of Ca^2+^ waves.

### pH_i_ gradients across confluent monolayers of myocytes induce [Na^+^]_i_ gradients

**Figure 7:**
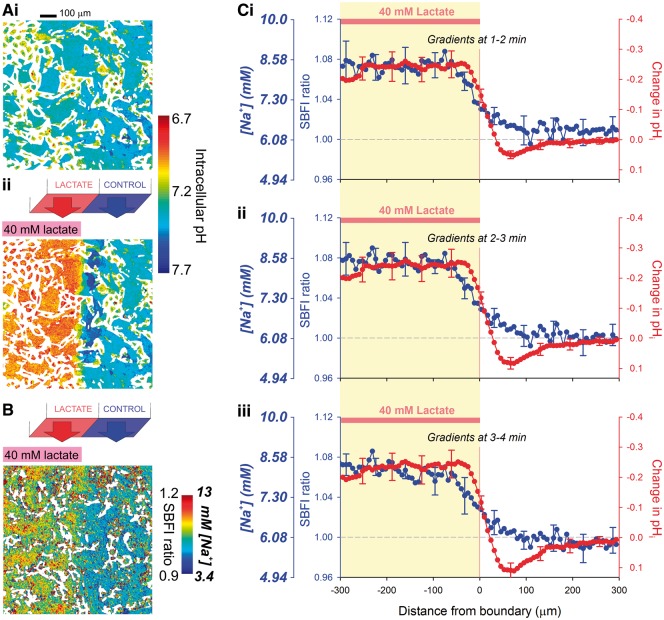
pH_i_ gradients are sustained in multicellular syncytial networks, and generate ${\text{Na}}_{i}^{\text{+}}$ gradients. (*A*) Exemplar cSNARF-1 fluorescence ratio map, calibrated in units of pH, of a confluent monolayer of neonatal rat ventricular myocytes under (*i*) control conditions and (*ii*) during regional superfusion with 40 mM lactate. (*B*) Exemplar fluorescence map showing [Na^+^]_i_ and SBFI ratio during regional superfusion with 40 mM lactate, normalized to starting levels. (*C*) SBFI ratio normalised to control conditions, absolute [Na^+^]_i_ (uniform exposure to lactate-free solution), and the change in pH_i_ (calculated from cSNARF-1 ratio) plotted across an axis perpendicular to the boundary between microstreams recorded at three different time-points during dual microperfusion. Measurements obtained from four monolayers. The rise in [Na^+^]_i_ in cells under the lactate-containing microstream is due to Na^+^ entry via NHE1, stimulated by the ensuing intracellular acidosis. The pH_i_ gradient across the inter-stream boundary is sharp due to slow H^+^ ion diffusion and rapid transmembrane H^+^-equivalent fluxes (lactate-H^+^ transport by MCT). The [Na^+^]_i_ gradient, in contrast, is shallower due to fast Na^+^ permeation through gap junctions and diffusion within the cytoplasm.

To explore whether spatial pH_i_ gradients can be established in multicellular cardiac structures, as well as in isolated cells, dual microperfusion was used to deliver a microstream containing 40 mM lactate in parallel to a lactate-free microstream over a confluent monolayer of neonatal rat ventricular myocytes. Under control conditions (i.e. uniform exposure to lactate-free solution), no pH_i_ heterogeneity was observed when imaging the cSNARF-1 fluorescence ratio (*Figure 7Ai*). However, when the monolayer was regionally superfused with 40 mM lactate (to represent the metabolic acidosis seen during myocardial ischaemia), a stable pH_i_ gradient of ∼0.3 units formed in the region of the solution boundary, of width ∼100 µm, equivalent to several neonatal cell-lengths (*Figure 7Aii*; cell-length is typically 20–40 µm). Cytoplasmic acidification in lactate-exposed cells activates NHE1 locally, which produces a rise in [Na^+^]_i_, detected using SBFI fluorescence.^30^ In the monolayers analysed in *Figure *7, cytoplasmic diffusion and gap junctional permeation of Na^+^ ions then resulted in a smooth gradient of elevated [Na^+^]_i_, ∼2.5 mM in magnitude, co-located with the pH_i_ gradient but extending up to 100 µm *beyond* the acidic zone. Note that, although SBFI fluorescence is modestly pH-sensitive,^31^ the ensuing SBFI response cannot be explained as an artefact generated by the underlying pH_i_ gradient, because the shapes of the pH_i_ and ${\text{Na}}_{i}^{\text{+}}$ profiles are not superimposable. The observations in monolayers are thus consistent with those made in isolated ventricular myocytes, showing that intracellular Na^+^ signalling can be stimulated downstream of a localised acidic domain.

## Discussion

We have demonstrated that acidosis powerfully stimulates arrhythmogenic Ca^2+^ waves. While this is consistent with earlier phenomenological descriptions,^5^ we have now quantified the relationship among [H^+^]_i_, [Na^+^]_i_, and Ca^2+^ waves, and demonstrated antagonistic control by $H_{i}^{\text{+}}$ and ${\text{Na}}_{i}^{\text{+}}$. Furthermore, since H^+^ and Na^+^ diffuse at different rates in myocyte cytoplasm, the control of Ca^2+^ wave generation becomes a complex spatio-temporal process. A key finding is that a localised source of intracellular acid inhibits Ca^2+^ waves in the immediate vicinity, but remotely stimulates them in distal, non-acidic regions, an effect mediated via the diffusible messenger, ${\text{Na}}_{i}^{\text{+}}$.

### Ca^2+^ wave frequency is controlled by inhibitory $H_{i}^{\text{+}}$ and excitatory ${\text{Na}}_{i}^{\text{+}}$ signals

During uniform acidosis, spontaneous Ca^2+^ wave initiation depends on a balance between the stimulatory effects of H^+^-activated Na^+^ influx via pH_i_ regulatory transporters such as NHE1, and the inhibitory effects of H^+^ ions on Ca^2+^ handling proteins, notably RyRs. Wave frequency is steeply stimulated by a rise of [Na^+^]_i_ compared with a more shallow inhibition by [H^+^]_i._ Thus, when NHE1 flux is enhanced, [Na^+^]_i_ rises, and waves can be profoundly stimulated. When, however, NHE1 flux is tempered, inhibitory effects of H^+^ can dominate, thus reducing Ca^2+^ wave frequency. This latter inhibitory effect has been suggested to be cardioprotective.^32^

A previous report concluded that intracellular acidosis caused only Ca^2+^ wave suppression,^6^ in contrast to our findings. This apparent discrepancy can be explained partly by the lower experimental temperature in the earlier study, which would decrease NHE1 activity,^33^ and by the briefer exposure to weak acid, which would limit any rise of [Na^+^]_i_. In our own work, we have clearly identified both ${\text{Na}}_{i}^{\text{+}}$-dependent stimulation and H^+^-dependent inhibition of Ca^2+^ waves.

We further demonstrate that increased [Na^+^]_i_ couples to Ca^2+^ waves via the modulation of sarcolemmal NCX, since the increase in wave frequency was prevented by pharmacological inhibition of this transporter. In principle, mitochondrial NCX could also make a contribution, however inhibiting mNCX provided no evidence for this. Instead, we confirm a cardioprotective role for mitochondria in buffering excess cytoplasmic Ca^2+^.^34^

### Rapidly diffusing Na^+^ ions couple local acidosis to remote Ca^2+^ wave stimulation

**Figure 8:**
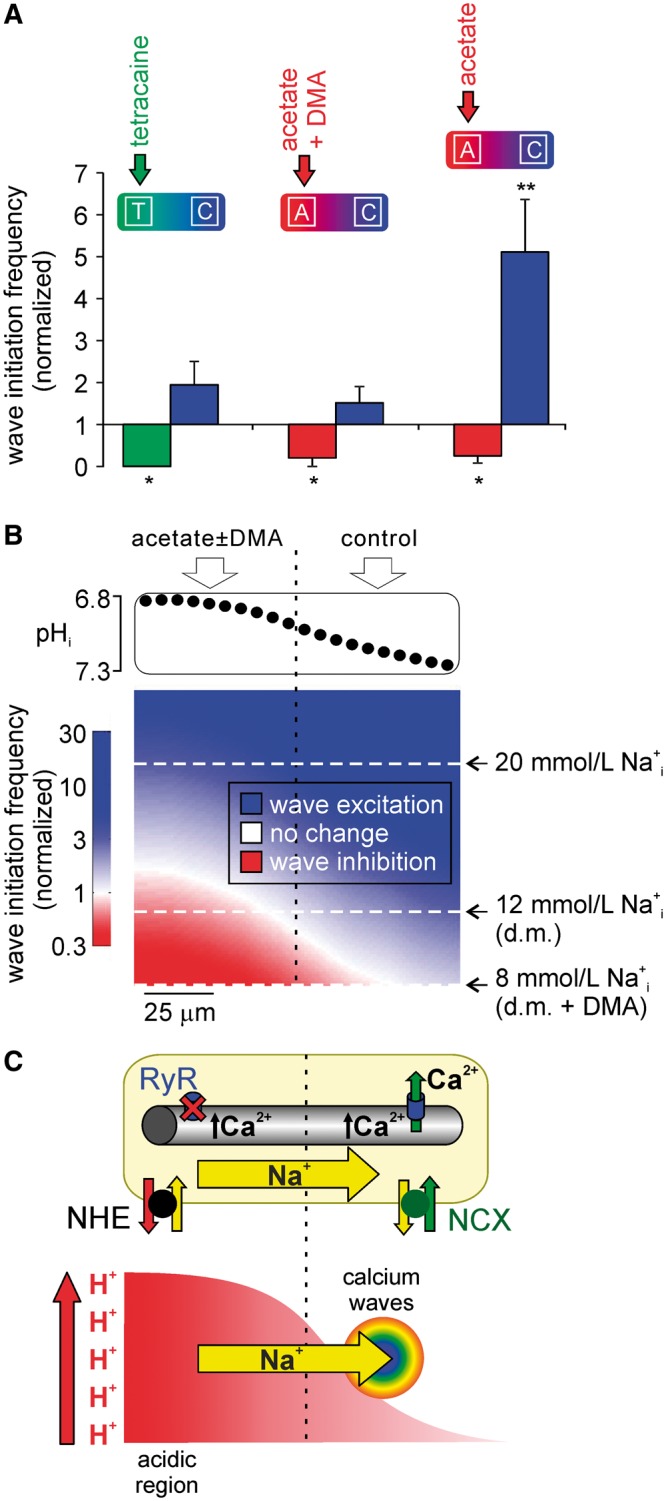
Local and remote pH-Ca^2+^ interactions. (*A*) Comparing spatial Ca^2+^ wave initiation data demonstrates that local inhibition of waves is NHE1-independent, and can be attributed to RyR inhibition, while remote stimulation only occurs in the presence of locally activated NHE1. For clarity, data from *Figures 4* and *5* replotted here to permit comparison. (*B*) The frequency of wave initiation depends on the balance between inhibition by H^+^ and stimulation by Na^+^. Top: the experimentally measured pH_i_ gradient illustrated here (*cf. Figure 4B*) was used in the model. Bottom: Results of a simulation based on the numerical relationships described in Supplementary material online, *Figures 1* and *2*. The model was used to predict wave initiation frequency (color-coded: red = wave inhibition, white = no change, blue = wave stimulation) during a pH_i_ gradient, for a range of [Na^+^]_i_ (*y*-axis, with representative values indicated). The simulation predicts (i) for resting [Na^+^]_i_ (8 mM, bottom white dashed line, i.e. when NHE is inhibited with DMA), waves are inhibited in the acidic region and remain at control frequency in the non-acidic region; (ii) for a rise in [Na^+^]_i_ comparable to the load during whole-cell acidosis^18^ (20 mM, top white dashed line), wave frequency is stimulated throughout the cell; (iii) for a modest rise in [Na^+^]_i_ (12 mM, middle white dashed line), as seen during an imposed pH_i_ gradient^18^, waves are inhibited in the acidic zone and stimulated downstream in non-acidic regions. d.m. = dual microperfusion. (*C*) Schematic of differential effects of pH_i_ non-uniformity on Ca^2+^ wave frequency. Local intracellular acidosis (represented in bottom part of schematic; *x-*axis is distance along cell) activates NHE1, leading to a rapid and global rise in [Na^+^]_i_ (fast diffusion—yellow arrow). This loads the SR throughout the cell, but waves preferentially emerge in the non-acidic microdomain due to the absence of H^+^ inhibition of RyRs.

Microdomains of pH_i_ are intuitively expected to affect local Ca^2+^ signals, under conditions such as vascular perfusion heterogeneity. The unexpected finding from our study is that an acidic microdomain can affect more than just local signalling; it drives downstream Ca^2+^ waves remotely, a phenomenon that depends on NHE1 activity in the acidic zone. For clarity, our results supporting this stimulatory mechanism have been amalgamated in *Figure 8A*.

One explanation for remote Ca^2+^ wave triggering is that the NHE1-driven increase in [Na^+^]_i_ raises luminal SR Ca^2+^ within the acidic zone (via ${\text{Na}}_{i}^{\text{+}}$-slowing of sarcolemmal Ca^2+^ efflux on NCX, and subsequent Ca^2+^-sequestration into the SR by SERCA). Elevated luminal Ca^2+^ might then diffuse throughout the whole SR, enhancing P_o_ for RyRs in non-acidic regions,^35^ thereby facilitating downstream Ca^2+^ wave generation. Measurements of SR Ca^2+^ diffusivity, however, are in the range of 9 to 60 µm^2^/s,^36^^,^^37^ values that predict a luminal transit time of about 6 and 1 min, respectively over a distance of 80 µm (mean distance between mid-point of microdomain ROIs within a single myocyte). Since the remote stimulatory effects on Ca^2+^ waves are already well established within 1 min of imposing a local acidosis, the contribution from diffusive re-distribution of luminal SR Ca^2+^ can, at best, only partially account for our observed results. Additionally, the re-balancing of flux between SERCA activity and passive leak would tend to return SR Ca^2+^ to physiological levels in regions away from the acidic source.

An alternative explanation is that rapid downstream diffusion of cytoplasmic Na^+^ ions, initially imported by NHE1 into the acidic microdomain, is the signal that raises SR [Ca^2+^] throughout the cell. This global stimulation by Na^+^ is then superimposed with local inhibitory effects of H^+^ in the acidic microdomain, leading to Ca^2+^ wave suppression in acidic zones, but wave initiation and clustering in downstream, non-acidic zones. Our recent measurements of high cytoplasmic Na^+^ diffusivity (∼680 μm^2^/s)^18^ are consistent with the time-frame of our Ca^2+^ wave responses.

To test the quantitative feasibility of the above hypothesis, a simple model was constructed (see *Figure 8*), based on the [Na^+^]_i_- and [H^+^]_i_-dependencies of Ca^2+^ wave frequency (*Figure 2Ai, Aii*). This was then used to predict the frequency of Ca^2+^ waves during imposition of a local acid load (*Figure 8B*). In the simulation, if [Na^+^]_i_ is restrained at resting levels of ∼8 mM (analogous to NHE1 inhibition), the model predicts that wave frequency is unchanged in areas of normal pH_i_, but inhibited in the acidic microdomain. If [Na^+^]_i_ is allowed to rise uniformly to around 20 mM, comparable to that observed under whole-cell acidosis (*cf*. *Figure 1Aiii*), the model predicts Ca^2+^ wave stimulation globally. Thus, regardless of the pH_i_ gradient, a significant ${\text{Na}}_{i}^{\text{+}}$ overload now promotes stimulation of waves in all cellular regions. However, the model prediction is radically different for an *intermediate* [Na^+^]_i_-rise to 12 mM. This rise is comparable to that measured experimentally during imposition of the pH_i_ gradient^18^ (note that, because of rapid diffusion, this [Na^+^]_i_-rise is near-uniform throughout the cell^18^). Under these conditions, the model predicts an inhibition of Ca^2+^ waves in the acidic microdomain and stimulation in the more alkaline microdomain (*Figure 8B*). Quantitative analysis therefore supports the mechanistic interpretation of our data.

We conclude that rapid spread of cytoplasmic Na^+^ ions, resulting in a modest, global rise of [Na^+^]_i_, is responsible for remote triggering of Ca^2+^ waves in regions where the pH_i_ is normal. Although [Na^+^]_i_ will also be raised at the source of acid, the superimposed inhibitory effects of H^+^ ions will dominate in that region. Our data suggest that local Ca^2+^ wave suppression is, at least in part, a result of RyR inhibition by H^+^ ions, on the basis that, in separate experiments, regional acidosis also suppresses local spark frequency.

### Ca^2+^ waves organized by spatial H^+^ and Na^+^ signals: cellular consequences

The above data and modelling indicate that spatial domains for H^+^ and Na^+^ will control both the site and frequency of spontaneous Ca^2+^ waves within a ventricular myocyte. Spatial pH_i_ non-uniformity will also grade wave propagation velocity, which will grade the magnitude of any consequent DAD.^38^ Because of low $H_{i}^{\text{+}}$ mobility, myocytes readily develop non-uniformity of pH_i_ during enhanced sarcolemmal H^+^ flux, compounded by the spatial distribution of H^+^-transporters, with NHE1 predominantly located at the lateral sarcolemma and intercalated discs.^21^ As a result, NHE1 activation results transiently in both longitudinal and radial pH_i_ gradients.^21^^,^^39^ Under conditions of Ca^2+^ overload, this pH_i_ pattern predicts preferential Ca^2+^ wave emergence in less acidic sub-sarcolemmal regions. This will influence the spatial distribution of excitation-contraction coupling within the cell, and may also modulate the gating of connexin channels at gap junctions.^9^ At present, in an isolated cell, it would be experimentally challenging to map Ca^2+^ waves to such dynamic pH_i_ gradients, but our current observations with larger, stable pH_i_ gradients show clear spatial wave initiation and clustering at the alkaline border of an acidic zone.

In our previous study, regional acidosis was found to influence *globally* the amplitude of the CaT within a ventricular myocyte, via rapidly diffusing ${\text{Na}}_{i}^{\text{+}}$, which co-ordinates the magnitude of electrically evoked SR Ca^2+^ release throughout the cell. This response suggested a mechanism for spatially unifying the contractile signal in the face of pH_i_ heterogeneity.^18^ In the present work, however, we find that the inhibitory and stimulatory effects of a local acidosis on Ca^2+^ wave frequency remain spatially separated. While this is reminiscent of the effect of local acidosis on diastolic [Ca^2+^],^24^ the mechanisms are dissimilar, since the diastolic effects are independent of changes in Na^+^, whereas remote wave stimulation is entirely dependent on the H^+^-driven rise in ${\text{Na}}_{i}^{\text{+}}$. The apparent paradox in the difference in spatial behaviour between Ca^2+^ waves and CaTs can be explained by considering the difference in nature of the events. CaT amplitude is a graded, continuous variable, and so we see a graded influence of [Na^+^]_i_ and [H^+^]_i_. In contrast, Ca^2+^ wave initiation is an all-or-none event. Although waves are, again, H^+^- and Na^+^-sensitive, we see a binary effect, dependent on the balance between the two probability distributions of H^+^-dependent inhibition and Na^+^-dependent stimulation. This difference between CaTs and Ca^2+^ waves means that, when pH_i_ is spatially heterogeneous, CaT amplitude can be fine-tuned in different regions of the cell, but the pH-dependent thresholding of wave initiation can result in spatially separated effects with wave suppression in acidic zones and wave triggering in non-acidic zones.

### Ca^2+^ waves organized by spatial H^+^ and Na^+^ signals: role in ischaemic myocardium?

Accumulation of metabolic weak acids such as CO_2_ and lactate is well documented within regionally ischaemic areas of myocardium,^40–42^ and this accumulation is expected to generate large and stable pH_i_ gradients across borderzones. In experimental models, gradients of extracellular myocardial pH can be as steep as 0.8 units, expressed over a few myocyte lengths.^42^ Indeed, here we have shown that a multicellular neonatal preparation can sustain a steep pH_i_ gradient across several cell-lengths, and that this induces an intercellular ${\text{Na}}_{i}^{\text{+}}$ gradient (*Figure 7*). The question is whether myocardial pH_i_ gradients will remotely trigger Ca^2+^ wave initiation in a way comparable to that observed in single myocytes. In the electrically coupled myocardium, because $H_{i}^{\text{+}}$ and ${\text{Na}}_{i}^{\text{+}}$ diffusivities differ substantially (100 μm^2^/s^20^ vs. 680 μm^2^/s^18^), the resulting myocardial spread of $H_{i}^{\text{+}}$ will be more restricted than that for ${\text{Na}}_{i}^{\text{+}}$. Consequently, any H^-^ dependent inhibition of aberrant Ca^+^-signalling within the ischaemic zone should also be spatially restricted, coupled with a wider spatial *stimulation* by Na^+^ ions, as illustrated schematically in *Figure 8C*. Propagation of Ca^2+^ waves through myocardial gap junctions has a high failure rate,^43^^,44^ while junctional H^+^ permeation is slow and requires chaperoning by carrier-molecules.^20^ In contrast, Na^+^ ions readily permeate gap junctions,^45^ which remain open even at relatively acidic pH_i_.^46^ Thus, in a regionally ischaemic myocardium, there is potential for a spatially defined borderzone of vulnerability, characterized by only modest acidosis, but an elevated [Na^+^]_i_ that is fuelled by Na^+^ diffusion from more acidic (ischaemic) zones (*Figure 8C*). The elevated [Na^+^]_i_ may contribute to the Ca^2+^ waves and arrhythmias that have been observed consistently at borderzones.^47^^,^^48^

Recent studies^47^^,^^49–51^ demonstrate that Ca^2+^ waves are most likely to be arrhythmogenic under conditions of local heterogeneity^50^ that favour the synchronous emergence of waves within a specific area of tissue. It is therefore tempting to suggest that pH_i_ non-uniformity in the myocardium^52^ may provide an important substrate for such synchronous Ca^2+^ waves. The arrhythmogenic risk will also be increased by the enhanced wave propagation velocity induced by acidosis. While many factors, other than H^+^ ions, also contribute to arrhythmogenic activity in ischaemia,^53^ the principle of remote H^+^-stimulation of Ca^2+^ waves, established here in our single cell studies, now merits experimental evaluation in the regionally ischaemic myocardium.

## Conclusions

We have shown that, in ventricular myocytes, H^+^ ions inhibit Ca^2+^ waves, via inhibition of Ca^2+^-handling proteins such as RyRs, but they also stimulate waves by activating Na^+^ influx on NHE1. The steep dependence of Ca^2+^ wave initiation on [Na^+^]_i_ often results in overall stimulation. When spatial pH_i_ heterogeneity is introduced into a myocyte, we find that acidic zones remotely trigger Ca^2+^ waves in non-acidic zones. This is explained by the different-sized spatial domains for elevated inhibitory [H^+^] and stimulatory [Na^+^], predicted by their different ionic diffusivities. As well as providing a stimulus for aberrant Ca^2+^ signalling in acidic myocytes, such spatial signalling may also have relevance in regional myocardial ischaemia, where the formation of pH_i_ gradients across groups of myocytes is likely. Remote H^+^-triggering of Ca^2+^ waves emphasizes the importance of Na^+^ as a spatial messenger in the heart.

## Supplementary material

Supplementary material is available at *Cardiovascular Research* online.

**Conflict of interest**: none declared.

## Funding

This work was supported by the British Heart Foundation (Programme Grant to RDVJ; RG/08/016 and RG15/9/31534, CRE Travelling Fellowship to RDVJ and MB); the Wellcome Trust (PhD Studentship to RDVJ and EM); and the Royal Society (University Research Fellowship to PS).

## Supplementary Material

Supplementary Data
