# Supplementary material for: Regional acidosis locally inhibits but remotely stimulates Ca2+ waves in ventricular myocytes
Source: Cardiovasc Res. 2017 Feb 21;113(8):984–95. doi: 10.1093/cvr/cvx033 (PMC5852542; doi:10.1093/cvr/cvx033)
Supplement: Supplementary Data [file cvx033_supp.zip › 3- FINAL (Revised RVJ ) Supplement 270116.docx]

**Supplemental material**

**Regional acidosis locally inhibits but remotely stimulates Ca^2+^ waves in ventricular myocytes**

Kerrie L. Ford, Ph.D.^1^, Emma L. Moorhouse, D.Phil.^1^, Mario Bortolozzi, Ph.D.^1,2^, Mark Richards PhD.^1^, Pawel Swietach, D.Phil.^1^, Richard D. Vaughan-Jones, Ph.D.^1*^

(1) Burdon Sanderson Cardiac Science Centre, Department of Physiology, Anatomy and Genetics, Oxford, UK

(2) Department of Physics and Astronomy “G. Galilei”, University of Padua, Padua, Italy

**Supplemental Materials and Methods**

**Ventricular myocyte isolation**

All procedures were performed in accordance with Directive 2010/63/EU of the European Parliament, UK Home Office and local guidelines. Adult rat ventricular myocytes were isolated from 300 g male Sprague-Dawley rats (Charles River, UK), killed using an approved Schedule 1 method (concussion followed by cervical dislocation). Cells were separated as previously described[^1^](#_ENREF_1). Briefly, hearts were retrogradely perfused using a modified Langendorff apparatus, initially with modified Krebs-Henseleit (K-H) solution plus 5 U/ml heparin, bubbled with 100% O_2_, before switching to modified K-H solution plus 300 U/ml collagenase type II (Worthington), and 0.3 U/ml protease type XIV (Sigma-Aldrich), for 10 minutes. After digestion, the ventricles were removed and mechanically disrupted to facilitate release of myocytes. Extracellular calcium was gradually restored to a final concentration of 1 mM. Following isolation, cells were maintained in modified K-H solution and used within 8 hours.

Neonatal rat ventricular myocytes were isolated from 1 day old Sprague-Dawley rat pups (Charles River, UK), killed using an approved Schedule 1 method (cervical dislocation). Hearts were excised, the ventricles dissociated, and subjected to 4 rounds of serial digestion using collagenase (Roche) and pancreatin (Sigma). Digestion was halted by adding neonatal calf serum to 10%. Cells were resuspended in high glucose DMEM supplemented with M199 (20%), horse serum (10%), neonatal calf serum (5%) and penicillin/streptomycin, and preplated for 2 hours at 37ºC to allow fibroblasts to settle. The supernatant containing the myocytes was removed and the myocytes plated onto coverslips coated with fibronectin. After 24 hours in culture the medium was removed and replaced with fresh DMEM supplemented with M199 (20%), horse serum (5%), neonatal calf serum (0.5%) and penicillin/streptomycin. After 48h in culture, cells were beating spontaneously. Cells were serum-starved for 24 hours prior to experiments.

**Solutions**

Modified Krebs-Henseleit solution (mM): 120 NaCl, 4 KCl, 10 HEPES, 11 glucose, 1.2 MgCl_2_, 20 taurine, 2 NaH_2_PO_4_, 2.5 pyruvate, pH adjusted with 4 M NaOH to 7.4 at 37°C. Normal Tyrode (mM): 135 NaCl, 4.5 KCl, 20 HEPES, 11 glucose, 1 MgCl_2_, 1 or 5 CaCl_2_, 1 probenecid, pH adjusted with 4 M NaOH to 7.4 at 37°C. Intracellular acidosis: Normal Tyrode solution contained 20, 40 or 80 mM sodium acetate, or 40 mM sodium L-lactate. Intracellular alkalosis: Normal Tyrode solution contained 20 mM trimethylamine. In acetate, lactate or trimethylamine-containing solutions, an equimolar amount of NaCl was removed to compensate osmolarity. Acetate anions are known to bind free Ca^2+^, therefore in acetate-containing solutions total CaCl_2_ added was increased by 25% to obtain the desired [free Ca^2+^]. All experiments were performed at 37°C.

**Intracellular pH measurements**

Cells mounted in a superfusion chamber on a coverslip coated with poly-*L*-lysine were imaged using a Leica SP5 inverted confocal microscope. To measure pH_i_, cells were loaded with 10 μM AM-esterified carboxy-seminaphthorhodofluor-1 (cSNARF-1, Life Technologies) for 10 minutes at room temperature. cSNARF-1 was excited at 514 nm and emitted fluorescence collected at 580 nm and 640 nm, and the 580/640 nm fluorescence ratio was calculated. Intracellular calibration of cSNARF-1 was performed using the nigericin method[^1^](#_ENREF_1) to derive values for intracellular pK_a_, R_max_ and R_min_.

**Intracellular calcium wave measurements**

Cells were loaded with 18 μM AM-esterified fluo-3 (Life Technologies) for 5 minutes at room temperature, then washed and allowed to de-esterify for a further 5 minutes. The dye was excited at 488 nm and emitted light collected between 510-580 nm. Calcium waves were imaged in linescan (*xt*) mode, scanning parallel to the longitudinal axis of the cell at 400 lines per second.

A quiescent myocyte was selected, before switching to Tyrode’s solution containing 5 mM CaCl_2_, to induce a modest calcium overload. Control waves were recorded for 60 seconds, before switching to the test solution and recording for a further 120 seconds. To induce an intracellular acidosis, cells were superfused with solutions containing 20, 40 or 80 mM sodium acetate. Intracellular alkalosis was induced by superfusion with solutions containing 20 mM trimethylamine.

All subsequent analysis was performed on the raw images. For presentation purposes, linescan images had the background subtracted, followed by generation of a pseudoratio F/F_0_ image. F_0_ was calculated as the mean fluorescence across the cell for the first second of imaging.

**Intracellular calcium spark measurements**

Cells were loaded with 18 μM AM-esterified fluo-3 (Life Technologies) for 5 minutes at room temperature, then washed and allowed to de-esterify for a further 5 minutes. The dye was excited at 488 nm and emitted light collected between 510-580 nm. A quiescent myocyte was selected, and calcium sparks were imaged in linescan (*xt*) mode, as for calcium waves. Sparks were recorded under control conditions (NT, 1 mM CaCl_2_) for 60 seconds, before switching dual microperfusion (microstream A: NT, 1 mM CaCl_2_; microstream B: 80 mM acetate, 1 mM free Ca^2+^) and recording for a further 120 seconds.

**Generating intracellular pH gradient (dual microperfusion)**

A pH_i_ gradient was induced using a square-bore double-barreled micropipette to release two parallel microstreams of solution, perpendicular to the cell[^2^](#_ENREF_2). One solution stream contained Tyrode with 5 mM CaCl_2_, the other 80 mM acetate (with 5 mM free Ca^2+^). One solution stream also contained 10 mM sucrose to enable visualization of the position of the solution boundary. In linescan experiments, the position of the microstream boundary along the cell was recorded in *xy* mode before and after each experiment.

**Data analysis**

Results are expressed as mean±SE. Statistical significance was tested using paired Student’s *t* test or nested ANOVA, according to the experimental protocol, and *P* = 0.05 was used as the confidence limit. Where relevant, the Holm correction for multiple testing was applied. Significance is denoted as * for *P*<0.05, ** for *P*<0.01, *** for *P*<0.001.

Wave propagation velocity was calculated from linescan images using the angle of incidence of the wavefront, relative to the longitudinal axis of the cell.

Linescan recordings of Ca^2+^ sparks were normalised using a custom-built Matlab macro, followed by spark frequency analysis using the MFDA algorithm developed by Kong *et al*[^3^](#_ENREF_3). For presentation purposes, images of sparks were processed in the same way as linescans of Ca^2+^ waves (see above) and were subjected to Gaussian smoothing to reduce background noise.

**Simulating [Na^+^]_i_- and pH_i_-dependence of wave frequency**

The model simulates the inhibitory effect of intracellular H^+^ ions (H^+^_i_) and excitatory effect of intracellular Na^+^ ions (Na^+^_i_) on Ca^2+^ wave initiation frequency. Initiation frequency, *F*, was modelled as the product of a Na^+^_i_-dependent function, *f*, and a H^+^_i_-dependent function, *g*:

$$F\left( H_{i}^{+},{Na}_{i}^{+} \right)=f({Na}_{i}^{+})\times g(H_{i}^{+})$$

The inhibitory, H^+^_i_-dependent function *g* was derived from figure 2Aii. Under these conditions, [Na^+^]_i_ is constant, and therefore function *f* is equal to unity.

$$g\left( H_{i}^{+} \right)=\frac{c}{H_{i}^{+}}$$

The constant of proportionality *c* was determined by best-fitting to be 50.5. Knowing function *g*, it was possible to derive the excitatory function *f* from frequency data obtained under conditions where [Na^+^]_i_ increases at low pH_i_ (i.e. absence of NHE1 inhibitors; figure 1Aiv). The Na^+^-dependence could be described by the following function of normalized [Na^+^]:

$$f({Na}_{i}^{+})=a\times exp(b\times({Na}_{i}^{+}-1)$$

Constants *a* and *b* were inferred from the excitatory effects of acidosis on frequency (figure 1Aiv), corrected for the concomitant inhibitory effect of H^+^ ions described by function *g* (since there is a small pH_i_ recovery during the Na^+^_i_ rise). [Na^+^]_i_ data were taken from [^4^](#_ENREF_4). By best fitting, *a*=16.3 and *b*=1.03. Figure 8 presents the results of the simulation, showing simulated wave frequency normalized to ‘resting’ frequency.

**Imaging neonatal rat ventricular cardiomyocytes**

Confluent cell monolayers were loaded with either cSNARF-1 (see methods for adult ventricular myocytes) or SBFI (10µM, 60 minutes loading in the presence of 0.1% pluronic acid F-127 at 37°C). Cells were recorded using a Leica SP5 inverted confocal system under control conditions with superfusion of normal Tyrode, followed by regional superfusion with 40mM lactate using the dual microperfusion device, to induce a pH_i_ gradient across the monolayer. Settings and calibration for cSNARF-1 imaging were the same as for adult ventricular myocytes. SBFI was excited at 355nm and emission was measured ratiometrically at 440±20nm and 565±15nm[^5^](#_ENREF_5). SBFI shows modest pH sensitivity[^6^](#_ENREF_6) with acidification producing an apparent decrease in measured [Na^+^]. Images were then normalized to the fluorescence ratio under resting conditions. SBFI was calibrated by incubating SBFI-loaded NRVM monolayers for 40 minutes with a solution containing 4.5mM KCl, 0.5mM EGTA, 1mM MgCl_2_, 11mM glucose and 0, 12.5 or 25mM NaCl. *N*-methyl D-glucamine was added as appropriate to titrate the osmolarity to 300mOsm. Solutions also contained 1mM strophanthidin, 2µM gramicidin and 30µM monensin to equilibrate intracellular and extracellular Na^+^. Monolayers were imaged at each [Na^+^] and this data used to produce a calibration curve (figure S4).

**Supplemental Results**

**S1. Some calcium waves convert to ectopic transients.** In some cells, Ca^2+^ waves trigger one or a train of ectopic CaTs. This is known to be due to a delayed afterdepolarisation (driven by the Ca^2+^ wave) triggering an ectopic action potential, which induces a whole-cell CaT. Panels show confocal linescans (AM-loaded fluo-3) of adult rat ventricular myocytes. Scale bars are 20 µm. Each panel represents 1 second of recording. Each panel is taken from a Ca^2+^-overloaded ventricular myocyte (5mM [Ca^2+^]_o_, n=4 cells). For example, top left-hand panel illustrates a spontaneous Ca^2+^ wave that triggers a full CaT, followed by two subsequent CaTs.

**S2. Intracellular acidosis stimulates Ca^2+^ waves, which can be attenuated by inhibiting NHE1 with DMA or cariporide.**

*Wave frequency data shown in figure 1 of main text are normalised. Here, in panels A and B, we show raw, non-normalised data, demonstrating the same phenomenon of wave stimulation or inhibition during intracellular acidosis* *(A)* Decreasing pH_i_ by 80mM acetate superfusion initially decreased, then increased wave frequency (raw data; n=18 cells/5 animals). Waves were counted in 10 second bins. *(B)* NHE1 inhibition reveals an underlying inhibitory effect of intracellular acidosis on Ca^2+^ wave frequency (raw data; n=12 cells/3 animals).

*In panels C and D, using normalised data, we show that cariporide exerts effects comparable to DMA. (C)* Inducing an intracellular acidosis with 80mM acetate stimulated Ca^2+^ wave initiation frequency. Wave frequency was calculated in 20s time bins and normalized to the average frequency during the control period. (n=9 cells) *(D)* Inhibiting NHE1 with 30μM cariporide converted stimulation of waves into inhibition. Since cariporide is a more specific and potent inhibitor of NHE1 than DMA, this suggests that possible inhibition of *I*_Na,P_  (the persistent Na^+^ current) by DMA does not play a role in wave inhibition (n=7 cells)

**S3. Intracellular acidosis alters multiple properties of Ca^2+^ waves.** *(Ai)* Intracellular acidosis increases diastolic [Ca^2+^], relaxation time constant acidosis (τ_control_=65.8±3.6 ms, τ_acetate_=82.8±4.1 ms, *P*=0.001), and also wave peak by 19% (*P*<0.0001; n=36 waves from 9 cells/3 animals)*. (Aii)* When NHE1 was inhibited, similar results were seen for diastolic [Ca^2+^] and relaxation time constant activity (τ_control_=68.5±3.7 ms, τ_acetate_=138±17.9 ms, *P*=0.0008), however wave peak decreased (by 19%, *P*<0.0001; n=24 waves from 6 cells/1 animal)*. (B)* Since the peak of the Ca^2+^ wave during acidosis was lower in DMA, the non-linear [Ca^2+^]-dependence of SERCA activity precludes direct comparisons of τ as a robust measure of overall SERCA function. To address this, wave relaxation rate, d(F/F_0_)/dt, was plotted versus F/F_0_. SERCA activity, described by this analysis, was shifted to higher F/F_0_ levels during acidosis, both in the presence (n=36 waves from 9 cells/3 animals) *(i)* and absence (n=24 waves from 6 cells/1 animal) *(ii)* of NHE1 activity (*P*<0.0001). Inhibiting NHE1 activity modestly increased the degree of right-shift of relaxation kinetics.

***Acidosis affects multiple properties of Ca^2+^ waves: additional detail*.** H^+^ displacement of Ca^2+^ from cytoplasmic buffers accounts for the observed rise in resting [Ca^2+^]_i_ during acidosis, as reported previously[^2^](#_ENREF_2), a rise that was DMA-insensitive (figure S3A). In contrast, the peak F/F_0_ fluorescence of Ca^2+^ waves *increased* during [H^+^]_i_ elevation, and this rise was attenuated (by ~25%) in the presence of DMA (figure S3A). The DMA did not greatly affect peak acidosis (see figure 1Aii, Bii), and the modest pH_i_ sensitivity of the Ca^2+^-fluorophore itself cannot explain the attenuation of F/F_0_ fluorescence[^4^](#_ENREF_4). Thus the magnitude of SR Ca^2+^ release during a wave is decreased by H^+^ ions, but enhanced by the associated NHE1 activity.

Elevating [H^+^]_i_ also caused an appreciable slowing of Ca^2+^ wave relaxation, an effect observed in the presence and absence of DMA (figure S3B). In rat ventricular myocytes, pH-sensitive relaxation of the electrically-evoked CaT is mediated mainly by SERCA[^7^](#_ENREF_7), so a similar mechanism for the pH-sensitivity of wave relaxation seems likely. Our observations on Ca^2+^ wave velocity and relaxation are thus consistent with inhibitory effects of H^+^ ions on Ca^2+^ buffers, RyRs and SERCA, which operate independently of [Na^+^]_i_. In contrast, Ca^2+^ wave amplitude, like wave frequency, is enhanced by NHE1 activity, reflecting a positive sensitivity to [Na^+^]_i_.

**S4. Properties of Ca^2+^ waves map on to pH_i_ microdomains.** *(A)* Average wave profile in the acidic (acetate) and non-acidic (control) microdomains during an imposed pH_i_ gradient. *(i)* Intracellular acidosis increases resting Ca^2+^, wave peak (18% increase, *P*=0.0337) and wave relaxation (τ_non-acidic_ = 140±10.8 ms, τ_acidic_ = 222±35.8 ms, *P* = 0.037) in the acidic microdomain compared with the non-acidic. n=18 waves from 6 cells/3 animals. *(ii)* When NHE1 was inhibited in the acidic microdomain, similar results were seen (wave peak: 28% increase, *P* = 0.006; τ_non-acidic_ = 224±18.5 ms, τ_acidic_ = 311±40.6 ms, *P* = 0.024). n=15 waves from 5 cells/2 animals. Paired Student’s *t*-test.

**S5. SBFI calibration in NRVM monolayers**. SBFI-loaded monolayers were exposed to solutions containing different [Na^+^] and strophanthidin, gramicidin and monensin to equilibrate intracellular and extracellular [Na^+^] (see Supplementary methods for further details).

**S6 Intracellular Na^+^ measurements: effect of changing extracellular [Ca^2+^]_._** We measured the rise of [Na^+^]_i_ in rat isolated ventricular myocytes (measured as a change in SBFI ratiometric fluorescence signal), during acidosis (addition of 80 mmol/L acetate *cf*. figure 1 of main paper), with 1 mmol/L or 5 mmol/L free [Ca^2+^]_o_ in the superfusate. The [Na^+^]_i_-rise towards its peak value, after imposition of intracellular acidosis, was not significantly different in the two situations. This is emphasized in figure S6A, where changes of intracellular SBFI fluorescence ratio were measured at fixed time intervals (up to 90s) after the switch to 80mM acetate superfusate. In addition, in further experiments, raising [Ca^2+^]_o_ from 1mM to 5mM exerted no effect on the resting level of Na^+^_i_ (in the absence of acetate, see figure 2B).

*(A).* Measuring the change of [Na^+^]_i_ in single isolated rat ventricular myocytes in response to 80 mmol/L acetate superfusion in presence of 1 or 5 mmol/L free [Ca^2+^]_o_ (blue and red bars, respectively). [Na^+^]_i_ was measured ratiometrically using SBFI and normalised to initial steady-state baseline fluorescence in the absence of acetate. Data were obtained by binning 6 seconds of data prior to timepoint shown. Myocyte [Na^+^]_i_ increased over the initial 90 seconds of acetate superfusion. This increase was not statistically different when the superfuste contained 1 mmol/L or 5 mmol/L [Ca^2+^]_o_ throughout the experiment (P=0.088, two-way ANOVA, n=20 cells/2 animals for 1 mmol/L [Ca^2+^]_o_ and n=26 cells /2 animals for 5 mmol/L [Ca^2+^]_o_ data).

(*B).* Resting [Na^+^]_i_ in rat ventricular myocytes showed no change when [Ca^2+^]_o_ was increased from 1 mmol/L (blue bar) to 5 mmol/L (red bars). [Na^+^]_i_ was measured ratiometrically using SBFI and expressed as absolute ratio. Data were obtained by binning 6 seconds of data prior to timepoint shown, superfusate [Ca^2+^]_o_ was changed at time = 0s. P=0.16, 1-way repeated measures ANOVA, n=20/2 animals.

**References**

1. Villafuerte FC, Swietach P, Youm J-B, Ford K, Cardenas R, Supuran CT, Cobden PM, Rohling M, Vaughan-Jones RD. Facilitation by intracellular carbonic anhydrase of Na^+^-HCO_3_^-^ co-transport but not Na^+^/H^+^ exchange activity in the mammalian ventricular myocyte. *The Journal of Physiology* 2013.

2. Swietach P, Youm J-B, Saegusa N, Leem C-H, Spitzer KW, Vaughan-Jones RD. Coupled Ca^2+^/H^+^ transport by cytoplasmic buffers regulates local Ca^2+^ and H^+^ ion signaling. *Proceedings of the National Academy of Sciences* 2013;**110**:E2064-E2073.

3. Kong CHT, Soeller C, Cannell MB. Increasing Sensitivity of Ca^2+^ Spark Detection in Noisy Images by Application of a Matched-Filter Object Detection Algorithm. *Biophysical Journal* 2008;**95**:6016-6024.

4. Swietach P, Spitzer K, Vaughan-Jones RD. Na^+^ ions as spatial intracellular messengers for co-ordinating Ca^2+^ signals during pH heterogeneity in cardiomyocytes. *Cardiovascular Research* 2015;**105**:171-181.

5. Baartscheer A, Schumacher CA, Fiolet JW. Small changes of cytosolic sodium in rat ventricular myocytes measured with SBFI in emission ratio mode. *J Mol Cell Cardiol* 1997;**29**:3375-3383.

6. Diarra A, Sheldon C, Church J. In situ calibration and [H+] sensitivity of the fluorescent Na+ indicator SBFI. *Am J Physiol Cell Physiol* 2001;**280**:C1623-1633.

7. Hulme JT, Orchard CH. Effect of acidosis on Ca^2+^ uptake and release by sarcoplasmic reticulum of intact rat ventricular myocytes. *Am J Physiol* 1998;**275**:H977-987.
