# Supplementary figures and images for: Regional acidosis locally inhibits but remotely stimulates Ca2+ waves in ventricular myocytes
Source: Cardiovasc Res. 2017 Feb 21;113(8):984–95. doi: 10.1093/cvr/cvx033 (PMC5852542; doi:10.1093/cvr/cvx033)

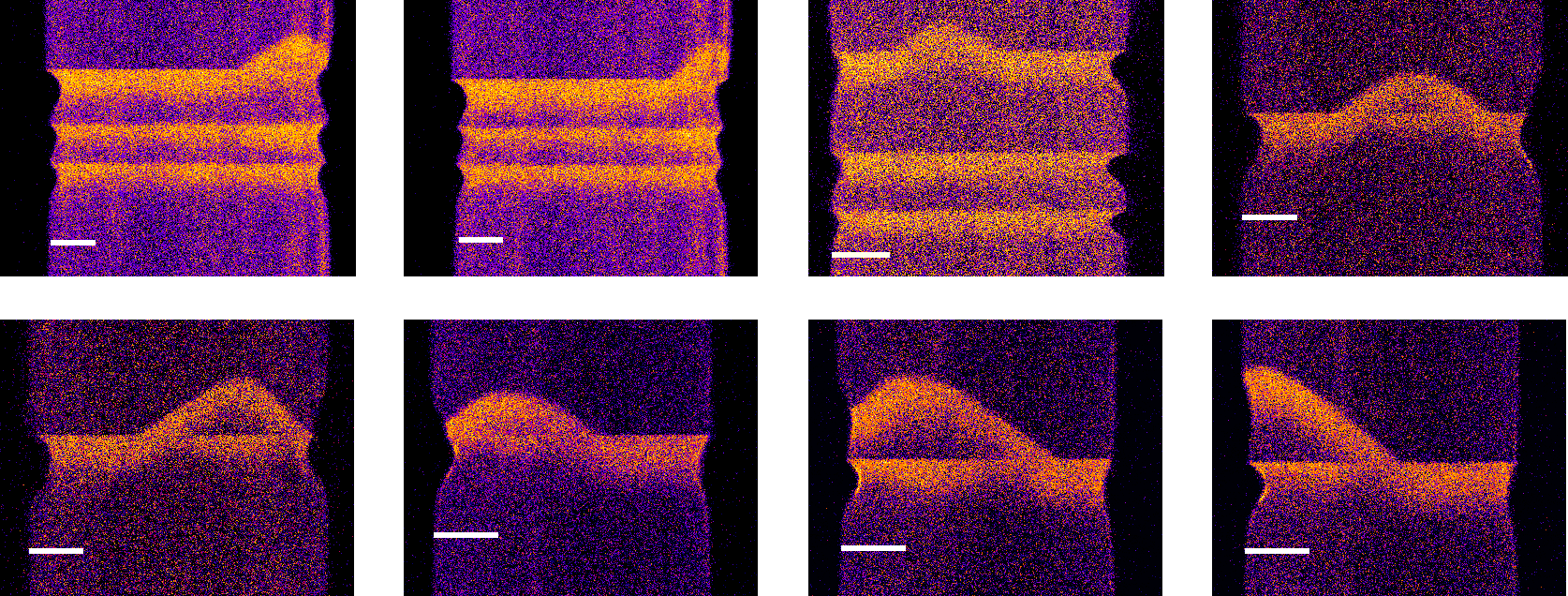

Supplement: Supplementary Data [file cvx033_supp.zip › Figure S1.tif]

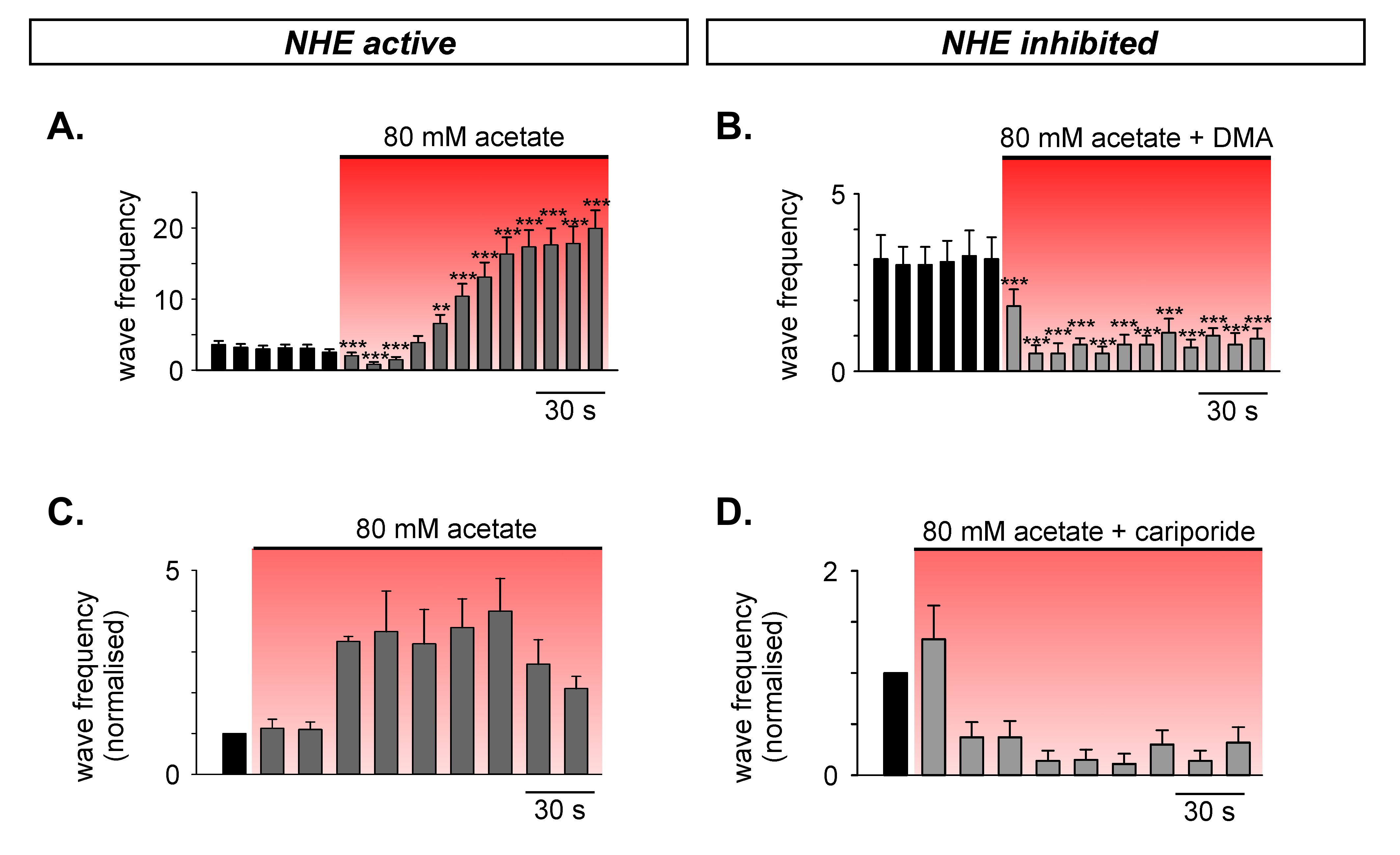

Supplement: Supplementary Data [file cvx033_supp.zip › Figure S2.tif]

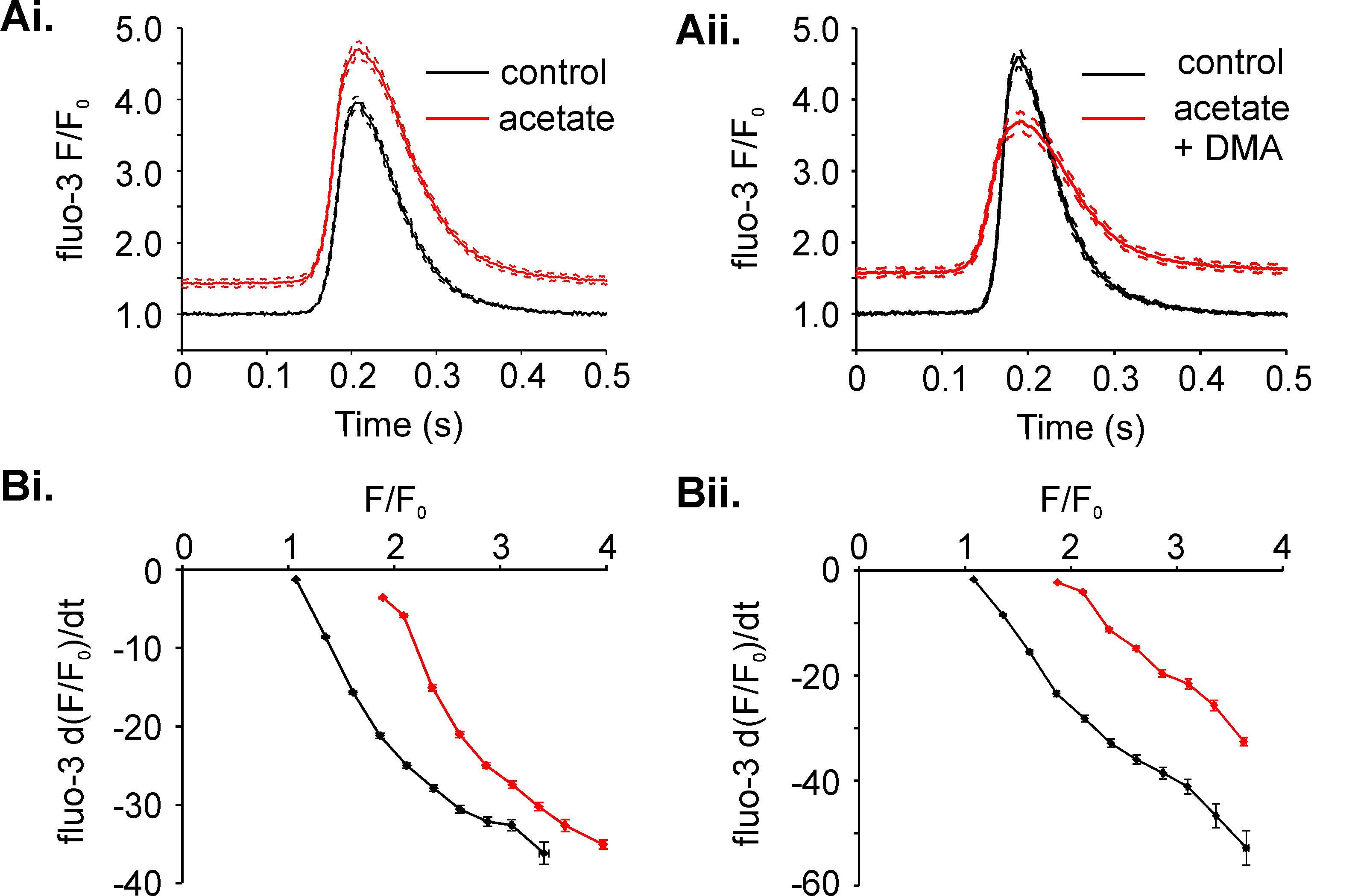

Supplement: Supplementary Data [file cvx033_supp.zip › Figure S3.tif]

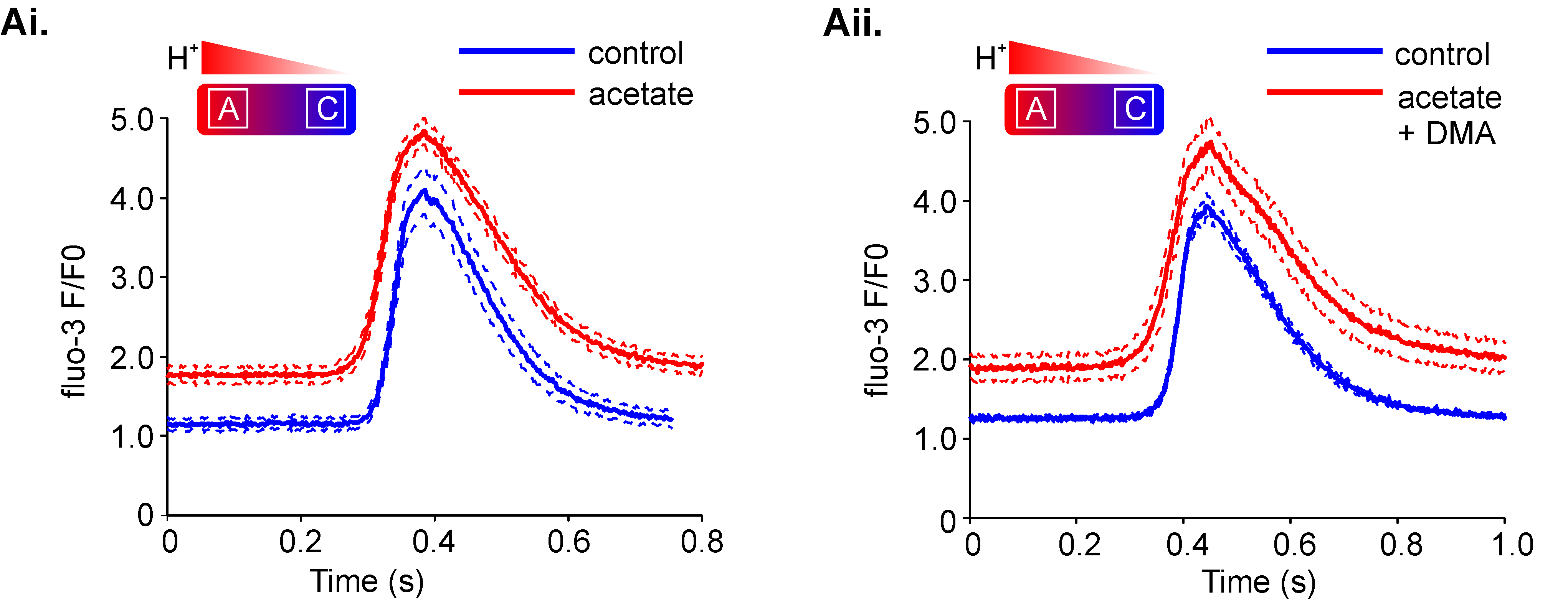

Supplement: Supplementary Data [file cvx033_supp.zip › Figure S4.tif]

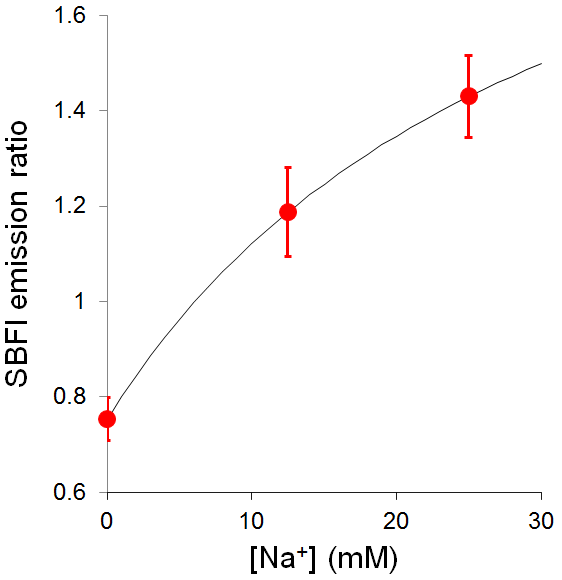

Supplement: Supplementary Data [file cvx033_supp.zip › Figure S5.tif]

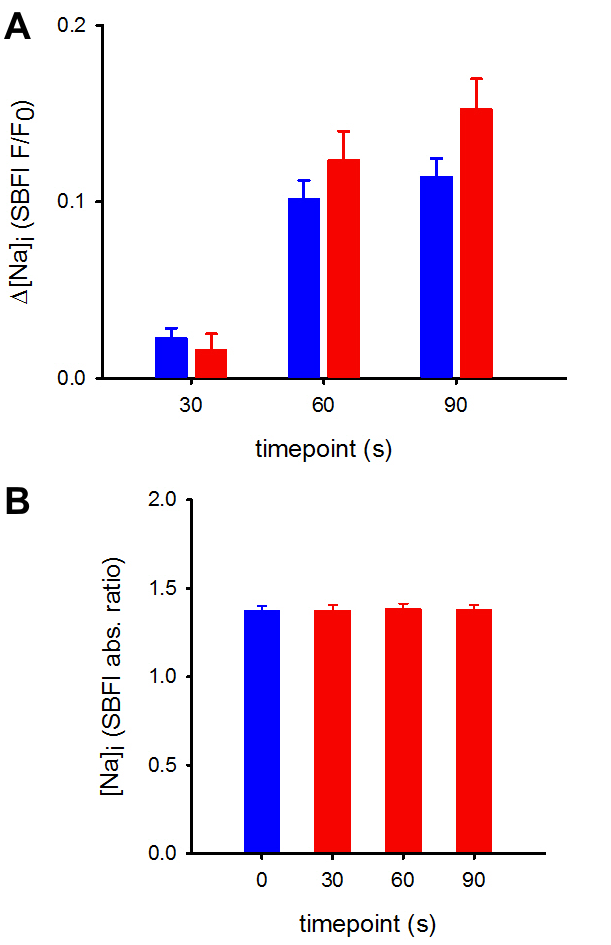

Supplement: Supplementary Data [file cvx033_supp.zip › figure S6.tif]
